# Correction: Evaluating the dispersibility of rGO prepared using a solvothermal method with DMF and its application as a CDI electrode material: recovery of sp^2^-hybridized carbon

**DOI:** 10.1039/d4ra90083a

**Published:** 2024-08-02

**Authors:** Junho Lee, Seonghyeon Ju, Chaehwi Lim, Jihoon Lee, Yeojoon Yoon

**Affiliations:** a Department of Environmental and Energy Engineering, Yonsei University Wonju 26493 Republic of Korea ljh20@yonsei.ac.kr sidzh20@naver.com w852741@yonsei.ac.kr battlela1@yonsei.ac.kr skyseeblue77@yonsei.ac.kr yajoony@yonsei.ac.kr +82-10-8993-0744

## Abstract

Correction for ‘Evaluating the dispersibility of rGO prepared using a solvothermal method with DMF and its application as a CDI electrode material: recovery of sp^2^-hybridized carbon’ by Junho Lee *et al.*, *RSC Adv.*, 2024, **14**, 22665–22675, https://doi.org/10.1039/D4RA03387F.

The authors regret that the title of this article was shown incorrectly in the original article. The corrected title is as shown above. The ESI has been updated online to reflect this change.

The Royal Society of Chemistry apologises for these errors and any consequent inconvenience to authors and readers.

